# How caregivers view patient comfort and what they do to improve it: a French survey

**DOI:** 10.1186/2110-5820-3-19

**Published:** 2013-07-01

**Authors:** Véronique Lombardo, Isabelle Vinatier, Marie-Lou Baillot, Vicenta Franja, Irma Bourgeon-Ghittori, Sandrine Dray, Sylvie Jeune, Chirine Mossadegh, Jean Reignier, Bertrand Souweine, Antoine Roch

**Affiliations:** 1 The Nurses’ board of the Société de Réanimation de Langue Française (SRLF), Paris, France; 2 Service de Réanimation Médicale, Hôpital Nord, Bd Pierre Dramard, 13915 Marseille cedex 20, France

**Keywords:** Intensive care unit, Comfort, Survey, Organization, Opinions

## Abstract

**Background:**

Intensive care unit (ICU) patients are exposed to many sources of discomfort. Most of these are related to the patient’s condition, but ICU design or how care is organized also can contribute. The present survey was designed to describe the opinions of ICU caregivers on sources of patient discomfort and to determine how they were dealt with in practice. The architectural and organizational characteristics of ICUs also were analyzed in relation to patient comfort.

**Methods:**

An online, closed-ended questionnaire was developed. ICU caregivers registered at the French society of intensive care were invited to complete this questionnaire.

**Results:**

A total of 915 staff members (55% nurses) from 264 adult and 28 pediatric ICUs completed the questionnaire. Analysis of the answers reveals that: 68% of ICUs had only single-occupancy rooms, and 66% had natural light in each room; ICU patients had access to television in 59% of ICUs; a clock was present in each room in 68% of ICUs. Visiting times were <4 h in 49% of adult ICUs, whereas 64% of respondents considered a 24-h policy to be very useful or essential to patients’ well-being. A nurse-driven analgesia protocol was available in 42% of units. For caregivers, the main sources of patient discomfort were anxiety, feelings of restraint, noise, and sleep disturbances. Paramedics generally considered discomfort related to thirst, lack of privacy, and the lack of space and time references, whereas almost 50% of doctors ignored these sources of discomfort. Half of caregivers indicated they assessed sleep quality. A minority of caregivers declared regular use of noise-reduction strategies. Twenty percent of respondents admitted to having non-work-related conversations during patient care, and only 40% indicated that care often was or always was provided without closing doors. Family participation in care was planned in very few adult ICUs.

**Conclusions:**

Results of this survey showed that ICUs are poorly equipped to ensure patient privacy and rest. Access by loved ones and their participation in care also is limited. The data also highlighted that some sources of discomfort are less often taken into account by caregivers, despite being considered to contribute significantly.

## Background

Patients are admitted to an intensive care unit (ICU) when their life is threatened by illness. An ICU stay is a source of both physical and psychological stress, during which invasive techniques are used and patients are exposed to specific conditions related to technical care, safety, and monitoring imperatives. The elements contributing to patient discomfort are multiple, related both to the patient’s condition and to design and organizational factors in the ICU. The major sources of patient discomfort have been identified as anxiety, pain, thirst, and sleep disturbance [1-4]. Discomfort may contribute to physical or psychological manifestations, such as depression and posttraumatic stress disorder (PTSD), which can affect quality of life after discharge from the ICU [5-7].

To help prevent patient discomfort in the ICU and to promote awareness of its importance, a consensus conference was convened in late 2009 by the French societies of adult and pediatric critical care [8]. Recommendations were made following this conference to improve patients’ , families’ , and caregivers’ experience of an ICU stay. In particular, improvements to the patient’s environment were recommended, as well as techniques likely to promote comfort and enhance communication with the patient. These recommendations were published [8,9]. Shortly after this publication, the survey presented in this article was designed and conducted by nurses’ board of the French-speaking society of intensive care. The results described represent a snapshot of caregiver opinions and practices before the recommendations become widely discussed and accepted. The survey was designed to determine the opinions of ICU caregivers on sources of patient discomfort and how they are dealt with in practice. Our survey also determined how design and organizational characteristics of ICUs contribute to patient comfort.

## Methods

### Questionnaire

The Nurses’ board of the French-speaking society of intensive care developed a questionnaire in April 2011. The questionnaire consisted of 52 closed-ended questions relating to: respondent’s role and characteristics; ICU characteristics in terms of design, equipment and organizational aspects potentially influencing patient comfort; how caregivers view sources of patient discomfort; and how patient well-being is considered by caregivers in practice. These questions were developed based on the recommendations of the consensus conference on critical care [8]. Most questions had four possible answers ranging from “never” to “always” for questions on practices or from “pointless” to “essential” for questions answered by opinions. Caregivers were asked to evaluate potential sources of discomfort on a 0 (not responsible for discomfort) to 10 (responsible for major discomfort) scale. The questionnaire was first tested on a panel of 30 doctors, nurses, and nurse’s aides. They were asked, in particular, whether all questions were clearly worded or open to misinterpretation. If misinterpretation was considered a potential problem, questions were reworded.

### Survey participation

In June 2011, an invitation to answer the questionnaire was sent by e-mail to 508 nurses and 1,250 physicians working in ICUs in French-speaking countries, using the society’s mailing list. Recipients also were encouraged to invite colleagues in the ICU to complete the questionnaire. The questionnaire was open to all caregivers (doctors, nurses, physiotherapists, nurse’s aides, psychologists). The questionnaire was designed to be directly and anonymously completed in its electronic format within 15 to 20 min. It was available online from June to September 2011. An e-mail reminder of the survey was sent in July and August 2011 to increase participation. The questionnaire was similar whatever the respondent’s role in the ICU. The respondent’s ICU could be identified during result analysis; this allowed data on architectural and organizational ICU characteristics to be confirmed by contacting the head nurse of the ICU. This was done for each ICU for which at least two caregivers had responded.

### Data analysis

Data were analyzed using SPSS 17.0 software (SPSS, Chicago, IL). Data were reported using descriptive statistics including frequency analysis (percentages) for categorical variables and mean ± standard deviation (SD) for continuous variables after checking their distribution. Statistical significance was examined using a Chi-square test or the Fisher exact test for categorical variables and Student’s *t* test for continuous variables. For most questions relating to practices, “often” and “always” responses were grouped, as were “never” and “rarely” responses. Similarly, for questions assessing caregiver opinions on practices “very useful” and “essential” responses were grouped, as were “pointless” and “not very useful” responses.

## Results

### Respondent characteristics

A total of 915 staff members from 264 adult and 28 pediatric ICUs completed the questionnaire in full (median (interquartile range) responses per ICU, 1 (1–2); Table 1). Of respondents, 55% were nurses. Notably, only 26% of respondents had already attended training on factors influencing patient’s well-being.

**Table 1:** Respondent characteristics

Total respondents	915
Age, yr (mean ± SD)	37 ± 10
Professional experience > 5 yr	527 (58)
Function	
Nurse	502 (55)
Day-shift	155 (31)
Night-shift	47 (9)
Day and night-shift	300 (60)
Physician	309 (34)
Nurse’s aide	80 (9)
Physiotherapist	20 (2)
Psychologist	4 (0.5)
ICU type	
Adult	853 (93)
Pediatric	62 (7)
Previously attended training on patient’s well-being	
All	242 (26)
Nurses	137 (27)
Physicians	66 (21)
Nurse’s aides	33 (41)*
Physiotherapists	3 (15)
Psychologists	3 (75)
Number of respondents per ICU	
1	213 (73)
2	35 (12)
3	34 (12)
≥4	10 (3)

Results are given as n (%) unless otherwise specified.

*p < 0.01 vs. nurses.

### ICU design and equipment

The characteristics of respondent’s ICUs are detailed in Table 2. Sixty-eight percent of ICUs had only single-occupancy rooms. This setup was more frequent in adult (72%) than pediatric ICUs (32%, *p* < 0.001). Natural light sources were present in each room in 66% of ICUs. Patients had access to a telephone in only 26% of ICUs and to a radio in 38%. Although a clock was present in every room in 68% of ICUs, the date was only visible in rooms in 11% of ICUs.

**Table 2:** ICU characteristics

	**All ICUs (n = 292)**	**Adult ICUs (n = 264)**	**Pediatric ICUs (n = 28)**
Tertiary teaching hospital	145 (50)	119 (45)	26 (93)*
French hospital	256 (88)	229 (87)	27 (96)
Mixed medical/surgical ICU	205 (70)	181 (69)	24 (86)
Beds per ICU (mean ± SD)	14 ± 8	14 ± 8	13 ± 5
*ICU design and equipment* (*available in each room*)			
Single-occupancy rooms only	200 (68)	191 (72)	9 (32)*
Natural light	194 (66)	176 (67)	18 (64)
Adjustable light intensity	230 (79)	209 (79)	21(75)
Call device	235 (80)	216 (82)	19 (68)
Phone	76 (26)	70 (26)	6 (21)
Television	173 (59)	152 (58)	21 (75)
Radio	112 (38)	97 (37)	15 (54)
Date	33 (11)	32 (12)	1 (4)
Time	198 (68)	183 (69)	15 (53)
*ICU organization*			
Patient to nurse ratio ≤ 2.5	145 (50)	126 (48)	19 (68)^†^
Patient to nurse’s aide ratio ≤ 4	181 (62)	170 (64)	11 (39)^†^
Full-time psychologist	24 (8)	15 (6)	9 (32)*
Visiting time (/day)			*
<4 h	129 (44)	129 (49)	0 (0)
4-12 h	111 (38)	108 (41)	3 (10)
13-23 h	9 (3)	5 (2)	4 (14)
24 h	43 (15)	22 (8)	21 (75)
Visits from children			
Strictly forbidden	16 (5)	13 (5)	3 (0)
With restrictions	240 (82)	217 (82)	23 (82)
Without restrictions	36 (12)	36 (12)	2 (7)
Gown required for visitors	162 (55)	141 (53)	21 (75)^†^
Pictures and personal objects allowed	280 (96)	253 (96)	27 (96)
Nonverbal means of communication	205 (70)	192 (72)	13 (46)^†^
Care activities often or always planned for family participation	30 (10)	13 (0.5)	17 (60)*
Nurse-driven analgesia protocol	124 (42)	115 (43)	9 (32)

Results are given as n (%) unless otherwise specified.

^*^p < 0.001 vs. adult ICUs; ^†^*p*< 0.05 vs. adult ICUs.

### ICU organization

The results presented in Table 2 reveal that only 50% of ICUs had a patient-to-nurse ratio in line with French recommendations (2 or more nurses for every 5 patients). Notably, more pediatric ICUs (68%) than adult ICUs (48%, *p* < 0.05) had a patient-to-nurse ratio ≤ 2.5. However, fewer nurse’s aides were employed in pediatric ICUs.

Visiting times tended to be more restricted in adult ICUs, with visiting times <4 h per day in 49% of these. In contrast, 75% of pediatric ICUs had a 24-h visiting policy (*p* < 0.001). Although only 15% of all ICUs combined had a 24-h policy, only 7% (n = 64) of respondents considered an unrestricted visiting policy to be pointless, whereas 64% (n = 582) considered it to be useful or essential to a patient’s well-being. This opinion was expressed more frequently by paramedics (66%) than doctors (59%, *p* < 0.05).

Family participation in care activities was planned almost exclusively in pediatric ICUs, with only 0.5% of adult ICUs adopting this approach. However, 27% (n = 223) of caregivers working in adult ICUs considered that family participation is or could be very useful or essential to the patient’s well-being, whereas only 9% (n = 75) considered it to be pointless. Children were allowed to visit in 95% of ICUs, with or without restrictions; 70% (n = 636) of respondents considered that visits from children were very useful or essential to patients’ well-being, and only 4% (n = 39) considered it to be pointless.

### Caregivers’ opinions on sources of discomfort and their practical management

Caregivers evaluated how different elements contribute to patient discomfort on a 0 to 10 scale (results are detailed in Table 3). For most questions in this part of the questionnaire, doctors gave a higher score than paramedics. When considering responses from both paramedics and doctors, patient discomfort was mostly attributed to anxiety, sleep disturbance, feeling restrained, noise, and pain. On the other end of the scale, lack of privacy or lack of moral support, light at night, missing loved ones and not being kept informed were considered less significant sources of discomfort.

**Table 3:** Sources of discomfort as evaluated by caregivers

**Paramedics**	**(n = 606)**	**Physicians**	**(n = 309)**
1. Anxiety	7.5 ± 1.9	1. Sleep disturbance	8 ± 1.6*
2. Feeling of restraint	7.3 ± 2.1	2. Anxiety	7.7 ± 1.8
3. Noise	7 ± 2	3. Noise	7.4 ± 1.8^†^
4. Sleep disturbance	7 ± 1.9	4. Feeling of restraint	7.4 ± 2.1
5. Feeling of dependence	6.4 ± 2.2	5. Pain	7.2 ± 2.6*
6. Pain	6.3 ± 2.6	6. Lack of space and time references	6.8 ± 2 *
7. Lack of space and time references	6.2 ±2.1	7. Thirst	6.7 ± 2.4*
8. Thirst	6.1 ± 2.2	8. Lack of information	6.6 ± 2.2*
9. Missing loved ones	5.8 ± 2.2	9. Feeling of dependence	6.5 ± 2.1
10. Lack of information	5.7 ± 2.4	10. Light at night	6.5 ± 2.1*
11. Light at night	5.6 ± 2.3	11. Missing loved ones	6.2 ± 2.1^†^
12. Lack of moral support	5.6 ± 2.3	12. Lack of privacy	6.2 ± 2.2*
13. Lack of privacy	5.4 ± 2.4	1. Lack of moral support	6.1 ± 2.2*

Discomfort sources were evaluated by caregivers on a sliding scale, from 0 (not responsible for discomfort) to 10 (responsible for major discomfort). Results are presented in decreasing order for paramedics (left column) and physicians (right column). Data are provided as mean ± SD. *p < 0.001 vs. same source for paramedics; ^†^*p* < 0.05 vs. same source for paramedics.

As was expected based on the scores given by caregivers, pain, discomfort related to position in bed, and anxiety were all taken into account as sources of discomfort as part of routine practice (Table 4). However, only 42% of ICUs used a nurse-driven protocol for analgesia despite 93% of respondents (n = 850) considering this type of protocol to be very useful or essential to improving patient’s comfort. Relaxation techniques often were or always were used by only 5% (n = 54) of respondents even though 80% (n = 712) considered them to be very useful or essential for patient’s comfort (86% of paramedics and 68% of physicians, *p* < 0.05).

**Table 4:** Proportion of caregivers routinely considering the different sources of discomfort

**Paramedics**	**(n = 582)**	**Physicians**	**(n = 309)**
1. Pain, based on usual scales	573 (98)	1. Pain, based on usual scales	301 (97)
2. Discomfort related to position in bed	571 (98)	2. Discomfort related to position in bed	266 (86)*
3. Anxiety	533 (92)	3. Anxiety	238 (77)*
4. Lack of privacy	480 (82)	4. Feeling of restraint	238 (77)
5. Thirst	468 (80)	5. Lack of information	230 (74)
6. Feeling of restraint	468 (80)	6. Sleep disturbance	210 (68)*
7. Sleep disturbance	458 (78)	7. Thirst	170 (55)*
8. Lack of information	444 (76)	8. Lack of space and time references	169 (55)*
9. Lack of space and time references	439 (75)	9. Lack of privacy	157 (51)*
10. Light at night	411 (71)	10. Light at night	142 (46)*
11. Noise	317 (55)	11. Noise	109 (35)*

Results are given in decreasing order for paramedics (left column) and physicians (right column). Only responses of paramedics directly involved in daily care (nurses and nurse’s aides) are presented. Data are provided as n (%). *p < 0.001 vs. same source for paramedics.

Some discrepancies were noted between scores and how sources of discomfort are dealt with in practice. In particular, noise, sleep disturbance and lack of space and time references were less frequently taken into account on a daily basis than would have been expected based on scoring (Table 4). In addition, only a minority of caregivers declared often or always using some means to reduce noise at night or to assess the quality and quantity of patient’s sleep (Table 5). Notably, more nurse’s aides than nurses indicated they often or always assessed noise-related discomfort (26 vs. 15% respectively, *p* < 0.01), or patients’ sleep quality (81 vs. 66% respectively, *p* < 0.01). No other significant differences were noted in practices and opinions between nurses and nurse’s aides.

**Table 5:** How caregivers deal with sources of discomfort

	**All (n = 891)**	**Paramedics (n = 582)**	**Physicians (n = 309)**
*Noise*			
Telephone in silent mode	19 (2)	15 (3)	4 (1)
Personalized alarm setting	361 (40)	294 (51)	67 (22)*
Personalized alarm sound level	187 (21)	158 (27)	29 (9)*
Relaxation time in a closed room	342 (38)	280 (48)	62 (20)*
Evaluation of noise-related discomfort	116 (13)	96 (16)	20 (6)*
Earplugs provided	34 (4)	33 (6)	1 (0)*
*Sleep*			
Sleep duration measured	418 (47)	326 (56)	92 (30)*
Patient asked about sleep quality	549 (62)	394 (68)	155 (50)*
Care planned in line with sleep	589 (66)	367 (63)	222 (72) ^†^
*Communication and how patients are perceived*			
Plan time for the patient to express his/her fears or anxieties	480 (54)	329 (57)	151 (49)^†^
Avoid talking to the patient	31 (3)	22 (4)	9 (3)
Consider the patient as a “subject of care” rather than as a “person”	131 (15)	87 (15)	44 (14)
Focus on security at the expense of patient comfort	420 (47)	248 (43)	172 (56)*
*Privacy*			
Talk to colleagues about unrelated matters in the presence of patients	174 (20)	145 (25)	29 (9)*
Provide care with doors closed	554 (62)	329 (57)	225 (73)*

Results are expressed as the proportion of caregivers who “often” or “systematically” use this practice. Data are provided as n (%). *p < 0.001 vs. paramedics; ^†^*p*< 0.05 vs. paramedics.

With regard to communication with the patient, half of survey respondents indicated that they often or always planned some time during consultations for the patient to express his/her fears or anxieties. Patients often were or always were perceived as a “subject of care” rather than as a “person” by only 15% of caregivers. Finally, almost half of respondents indicated that, when administering treatment, they often or always focus on safety rather than on patient comfort.

With regard to respecting patient privacy, although only a minority of caregivers admitted to often or always having conversations about unrelated topics with colleagues in the presence of patients (Table 5), only 17% (n = 150) reported never doing so. Approximately 40% indicated that care often was or always was provided without closing doors. Finally, more paramedics (51%) than doctors (39%, *p* = 0.001) often or always addressed the question of the patient’s well-being in written handover or medical records. During staff meetings, this question was addressed often or always by only 33% (n = 300) of caregivers (39% of paramedics vs. 31% of physicians, *p* = 0.1), whereas 12% (n = 33) of physicians and 17% (n = 105) of paramedics never addressed the question of patient’s well-being.

## Discussion

Our survey reveals that the design and organization of many French ICUs remain poorly adapted to promoting patient comfort. Caregivers responding to our survey consider that anxiety, sleep disturbance, feelings of restraint, noise, and pain are the most significant sources of patient discomfort. However, caregivers rarely take action to alleviate them.

Patients have identified anxiety, pain, thirst, and sleep disturbance as major sources of discomfort and stress during their ICU stay [1-4]. Our results indicate that caregiver’s identification of sources of discomfort at least partly overlaps with patient experience, with caregivers viewing pain and anxiety as the main sources of patient discomfort. Pain is a major source of discomfort, with half of the patients surveyed in previous studies reporting that they experienced pain during their ICU stay [1,2]. Because pain has been linked to delirium and PTSD [10,11], rapid, patient-tailored pain relief is strongly recommended. However, although almost all those responding to our survey routinely evaluated and treated pain, less than half the responding ICUs had a nurse-driven analgesia protocol. This type of protocol was nevertheless considered to be very useful or essential by almost all respondents.

According to our results, anxiety is considered on a daily basis by a large majority of caregivers. Nevertheless, only a small number of ICUs have a full-time psychologist, apart from pediatric ICUs, where their presence has been recognized as necessary for many years as part of support for sick children, parents, and staff. Additionally, some factors contributing to anxiety (such as lack of space and time references, sleep disturbance, lack of information, or missing loved ones) are insufficiently addressed. For example, in one-third of ICUs, patients did not have access to a clock, and only very few ICUs kept them informed of the date. Anxiety can lead to sleep disturbance, which is frequent in the hospital setting [12] and is reported as stressful by two out of three patients [2]. Despite this, only half of our respondents routinely evaluated patient’s sleep. Causes of sleep disturbance were frequently noted, such as multiple-occupancy rooms, no means to adjust light intensity, and limited efforts by many caregivers to reduce noise. Patient anxiety also can be promoted by a lack of information and an inability to communicate [1,13]. To help overcome this inability, nonverbal means of communication are recommended [14-16]. However, our results reveal that these methods are not used in one-third of our respondents’ ICUs. Finally, restricting visits from loved ones is also a source of patient anxiety and PTSD [10,13,17]. The French consensus conference [8] concluded that next of kin should be allowed to visit without time restrictions, in line with the needs of care and patients’ wishes; children also should be admitted as part of supervised access [18]. The results presented here reveal that one in two adult ICUs still have restrictive visiting policies. However, a 24-h visiting policy was advocated by a large majority of respondents. Interestingly, physicians were slightly more reluctant to adopt a liberal policy than paramedics even though a 24-h visiting policy was demonstrated to be favorably perceived by caregivers in units where it was tested [19]. Although it is not currently recommended, our results showed that a majority of ICUs require visitors to wear a gown. This could contribute to preventing visitors from feeling comfortable while visiting patients. A gown was required in as many ICUs with a 24-h visiting policy as ICUs with restricted visiting. This suggests that this practice is not directly linked to an overall policy of facilitating family’s access to ICUs.

In ICUs, noise levels have been extensively demonstrated to be above the World Health Organization recommendations [20,21]. In our survey, noise was considered as one of the main sources of patient discomfort. However, although a memory of irritating noises has been shown to be associated with the occurrence of PTSD [22] most patient-based studies did not rate noise as very stressful [1,2]. This possible overestimation of noise as a source of discomfort by caregivers could be because they consider noise to be the main cause of sleep disturbance whereas, in fact, anxiety and pain may be more to blame. It also may indicate that the noisy environment of many ICUs is more readily perceived by caregivers. Although noise levels could be readily modified by applying some simple strategies [23], few of those responding to our survey used methods to reduce noise or to assess how noise affects patient comfort.

The body often is exposed during care [24], and both patients and families indicate that privacy and confidentiality should be respected during care [25,26]. An adapted single-occupancy room favors privacy and confidentiality, allows families to participate in care and encourages closer relations with loved ones during an ICU stay [25,27]. In the present survey, one-third of ICUs did not have only single-occupancy rooms. We also found that family participation in care remains very rare in adult ICUs. A recent single-centre survey found that perception of participation in simple care, such as moistening of the oral cavity or hydrating the lips, was very favorably perceived by both caregivers and family [28].

The present study has some limitations. It is based on a survey of opinions and declared practices, rather than a practice audit. ICUs or caregivers were not preselected, and the proportion of ICUs from teaching hospitals represented is higher than the national average. Because of this, our results may not be perfectly representative of ICU policies and opinions in French-speaking areas. However, there was no significant difference in characteristics of caregivers from teaching and nonteaching hospitals. The response rate also was much higher for nurses than for physicians, which limits the validity of comparisons between these two groups. Moreover, this difference suggests that efforts must be made to motivate participation by doctors interested in the field of quality of care.

## Conclusions

This survey of caregivers in French ICUs reveals that efforts to improve patient well-being should be pursued. Not enough ICUs are designed to preserve patient privacy and to create a restful atmosphere. In addition, an organization encouraging access to loved ones and their participation in care has not been widely adopted. This survey also highlighted which sources of discomfort should be the focus of more attention from caregivers and gives indications for how patient comfort can be improved. For example, extending visiting hours, reducing noise and light at night, promoting nurse-driven analgesia protocols, improving communication through the help of psychologists, and addressing well-being issues during meetings should all be considered. Future surveys should evaluate other important points that have not been extensively addressed here, such as physical restraint, and should assess recently developed tools to improve patient well-being during and after ICU stay [9,28-30].

## Competing interests

The authors declare no conflict of interest that may inappropriately influence how results are presented in this paper.

## Authors’ contributions

Study design, data collection and analysis: VL, IV, MLB, VF, IBG, SD, SJ, SL, YM, FM, CM, JR, BS, and AR. Manuscript preparation: AR. All authors read and approved the final manuscript.
